# What evidence exists on the effectiveness of algae as biomonitors of pollution in estuaries? A systematic map protocol

**DOI:** 10.1186/s13750-025-00378-1

**Published:** 2025-11-13

**Authors:** Daniel Tremmel, Carla Carvalho, Túlio Silva, Jana Del Favero, Bruno Guides Libardoni

**Affiliations:** 1 Federal Fluminense University, Rio de Janeiro, Brazil; 2 Infinito Mare, São Paulo, Brazil

**Keywords:** Aquatic toxicology, Algae, Water pollution, Nature-based solutions, Biofiltration

## Abstract

**Background:**

Estuarine coastal regions play a critical role in global aquatic ecosystems, providing essential benefits such as diverse marine habitats, support for local economies through fisheries and tourism, and serving as important carbon stocks. Nonetheless, these invaluable, dynamic and complex habitats are under increasing threat from human-induced pressures, including pollution from agricultural runoff to sewage discharge, emphasizing the urgent need for innovative monitoring and mitigation strategies. Traditional biomonitoring methods involve the use of indicator species such as fish and benthic macroinvertebrates; however, these can be limited in their ability to detect pollution at an early stage. As a result, alternative monitoring strategies such as the use of algae have become increasingly popular due to their abundance sensitivity to changes in water quality. Previous research recognizes the capacity of various algae species to accumulate pollutants, thereby serving as reliable indicators of ecological stress and water contamination. Despite the growing acknowledgment of their potential, a comprehensive evaluation of the effectiveness of algae as biomonitors in estuaries remains without a systematic review. This map, therefore, seeks to synthesize existing knowledge on the applicability and reliability of algae for coastal environmental monitoring, aiming to highlight existing knowledge gaps for a future systematic review. By focusing on the utility of algae in estuarine contexts, this study aspires to provide a comprehensive overview of current practices and propose recommendations. Such an endeavor is crucial for directing future research, informing stakeholders, and guiding policy formulation towards more sustainable and effective environmental management of estuaries. This map aims to be a valuable resource for those involved in the management and preservation of estuarine environments, contributing to discussions on sustainable water management and ecological conservation.

**Methods:**

The Collaboration for Environmental Evidence Guidelines and Standards for Evidence Synthesis in Environmental Management will be followed to construct the systematic map. By using a tested search string consisting of English keywords and acronyms, we will look through two published databases (Scopus and Web of Science Core Collection) to find pertinent literature. Terms that describe the exposure (chemicals) and the population (algae in estuaries) will be combined in the search string. To this literature obtained so far, we will add more materials sourced from other search mechanisms. We will add to this body of literature with further material from Google Scholar and other internet searches, including sources in Portuguese. Next, adopting specified eligibility criteria, titles, abstracts, and full-texts will be analyzed one by one. A list of predefined variables will then be extracted from full-texts. A database containing all studies included in the map, along with coded metadata, will be generated. The evidence will be presented in a map report that includes text, figures, and tables. A matrix will be created to display the distribution and frequency of the included studies categorized by types of exposure and outcomes, aimed at identifying potential knowledge gaps and clusters.

**Supplementary Information:**

The online version contains supplementary material available at 10.1186/s13750-025-00378-1.

## Introduction

Biomonitoring estuaries is the process of assessing the health of estuarine ecosystems by monitoring the presence and abundance of certain organisms or pollutants. Estuaries are constantly subject to environmental impacts of human activities, such as fisheries, ship traffic, ports, industries, urban and domestic influence, which can affect the health and survival of the local biota [14, 16, 25]. Biomonitoring can help guide authorities regarding sewage management and ensure the evolutionary development of estuarine species. Anthropogenic synthetic compounds of organic nature, including microplastics, pharmaceuticals, pesticides, polychlorinated biphenyls (PCBs), and polycyclic aromatic hydrocarbons (PAHs), are frequently present in marine environments and have been detected in the global marine system [26]. These persistent organic pollutants (POPs) have direct and indirect effects on marine biota, including impacts on different marine and human health. While petroleum hydrocarbons are known to be highly toxic, causing habitat destruction, mass mortality, and a range of physiological impairments in marine life (reduced feeding, slow growth, respiratory problems, impaired locomotion) [4], it's crucial to recognize that many other anthropogenic pollutants share these damaging characteristics. Heavy metals, for example, can bioaccumulate in food chains, impacting organisms at various trophic levels, ultimately affecting humans [2]. Similarly, pesticides and herbicides, while often targeting specific organisms, can have cascading effects on entire ecosystems, disrupting sensitive ecological balances [20]. Therefore, our society’s dependence on petroleum derivatives and industrialized chemicals such as hormones and pharmaceutical products is already causing impacts on marine life and affecting seafood quality [3, 4, 19, 21, 24].

Algae utilize nutrients in water to photosynthesize, and their diversity and abundance can be used as an indicator of eutrophication levels [23]. Additionally, algae have been shown as biomonitoring agents to assess the presence and concentration of heavy metals and organic pollutants in water bodies. Algae are also used for bioremediation of wastewater due to their economical and easy-to-achieve culture on a large scale. Large-scale algae biomass production while treating sewage and recycling waste has been used synergistically to convert into methane, fertilizer, and reclaimed water [1, 5, 13]. Algae can also be used as a tool to evaluate potential adverse effects on aquatic biota by assessing their behavior in response to toxicity and ecological environmental stress [8, 15,17]. The use of algae as bio-indicators of pollution is particularly important in coastal ecosystems, where harmful algal blooms (HABs) are increasing due to anthropogenic nutrient enrichment derived from riverine and urban inputs. Algal responses to stressors caused by humans help diagnose stressors and establish targets for protection and restoration. They have thresholds that are particularly important for developing consensus for coastal management targets.

The stakeholders interested in this topic include environmental managers, researchers, policymakers, and industries. The effectiveness of algae as biomonitors of pollution in estuaries is crucial for developing sustainable and environmentally friendly practices in these industries and for protecting the health of marine ecosystems and human populations that depend on them [7, 9]. However, there is a lack of uniformity in the conclusions reached, and further research is required to evaluate the effectiveness of algae as biomonitors of pollution in estuaries [11, 22].

Algae stand out as effective biomonitors of pollution due to their sensitivity to a wide range of environmental stressors, their rapid response to change, and their widespread distribution in aquatic ecosystems. The theory of change for using algae as biomonitors of pollution considers both the accumulation of contaminants and the various responses of algal species to the contaminants present in water. It starts with the pollutants, such as petroleum hydrocarbons, heavy metals, pesticides, or pharmaceuticals, entering the estuary and being taken up by various algal species. Some species are more efficient at eliminating these contaminants than others, which leads to a selective pressure where sensitive species experience reduced growth or die-off due to bioaccumulation, while more tolerant species might thrive in their absence, leading to shifts in community composition. By analyzing both the concentration of contaminants in algal tissues and the changes in species abundance, a more comprehensive understanding of the ecological impacts of pollution is provided. This approach not only identifies the presence of contaminants in algal tissues but also reveals how pollution disrupts the natural balance of the ecosystem.

Compared to other organisms, algae offer several advantages: they are relatively easy to sample and cultivate, they exhibit a wide range of measurable responses to pollutants (from physiological changes to community shifts), and their short life cycles allow for quick detection of environmental change. Analyzing pollutant concentrations directly in algal tissues, rather than solely in the surrounding water, provides a more comprehensive and insightful approach to pollution monitoring. This is because algae can bioaccumulate pollutants to detectable levels even when water concentrations are low, offer insights into the bioavailable fraction of contaminants, and reflect historical exposure through time-integrated accumulation. Therefore, analyzing algal tissues enhances the sensitivity, relevance, and temporal scope of pollution monitoring efforts.

In terms of coastal management targets, establishing thresholds for algae, such as chlorophyll levels used in trophic state indices, can be a powerful approach. Exceeding thresholds, often linked to excessive nutrient loading, can signal a decline in water quality and potential harm to sensitive species. The presence of specific toxin-producing species can also provide clear warning signs of ecosystem degradation. These thresholds, often based on local background levels, regulatory standards, or ecological tipping points, are crucial for the development of timely management interventions to mitigate pollution and protect environmental health.

Climatic conditions and external stressors play a crucial role in shaping the results of pollution studies using algae as biomonitors. Different locations and study areas experience unique combinations of temperature, salinity, nutrient levels, and other environmental factors, all of which can influence the sensitivity and responses of algal communities to pollutants. For instance, algae in warmer waters might exhibit heightened sensitivity to certain contaminants or recover more slowly from pollution events. Similarly, the presence of other stressors, such as coastal development or invasive species, can interact with pollution, leading to complex and site-specific effects on algal communities that must be considered. Both water residence time and precipitation patterns interact to influence the availability of nutrients, which is a key factor driving algal growth and biomass accumulation. Understanding these dynamics is crucial for interpreting algal biomonitoring data and developing effective water resource management strategies.

Furthermore, the study's sampling methods are equally critical in influencing the results. The choice of sampling sites, frequency, and duration, as well as the methods used to collect and analyze algal samples, can introduce variability and bias and needs to be clear in the selected papers. For example, sampling only during a particular season might miss pollution events that occur at other times of the year, while inconsistent sampling techniques can lead to inaccurate assessments of algal responses. Therefore, careful consideration of both local environmental conditions and robust sampling methods is paramount for obtaining reliable and meaningful results in algal biomonitoring studies.

By understanding how different pollutants affect algae's biological and chemical composition, we can identify sensitive species and responses that can serve as reliable indicators, such as providing levels of ecosystem health. Therefore, mapping the literature looking for evidence of the impacts of pollution on algae provides a crucial background for developing effective biomonitoring strategies using these organisms. Given the increasing concern over pollution in estuarine ecosystems, this systematic map collates what evidence exists to evaluate the effectiveness of using algae as biomonitors.

### Stakeholders engagement

Embedded within the "RadiCal—Radiocarbono e Algas" PhD research project at Fluminense Federal University, in partnership with Infinito Mare and supported by CNPq, this protocol advances a systematic examination of chemical pollutants' impacts on coastal and estuarine algae. This research is a direct response to the urgent need for sustainable coastal management solutions, aligning closely with the United Nations Sustainable Development Goals (SDGs), particularly Goal 14—Life Below Water, which calls for the conservation and sustainable use of the oceans, seas, and marine resources. By meticulously investigating the complex dynamics between algae and chemical contaminants, the study aims to uncover critical insights for closing existing knowledge gaps. These findings will empower policymakers with science-based tools and strategies, fostering sustainable policies that protect and enhance marine biodiversity. This initiative champions an integrative approach to environmental stewardship, demonstrating a proactive commitment to global sustainability objectives and ensuring healthier marine ecosystems for future generations.

## Objective of the review

### Primary question

The main question of this review is: What evidence exists on the effectiveness of algae as biomonitors of pollution in estuaries? (what type of pollutant, algae, impacts, and mechanisms).

### Components of the primary question

*Population*: algal species in estuaries.

*Exposure*: types and concentration of natural and human-produced chemical substances.

*Comparator*: population not exposed to pollution; population prior to pollution exposure/ population exposed to different concentrations of pollution.

*Outcome*: All outcomes related to estuarine algae being used as biomonitors of pollution, from molecular to community levels.

**Fig. 1:**
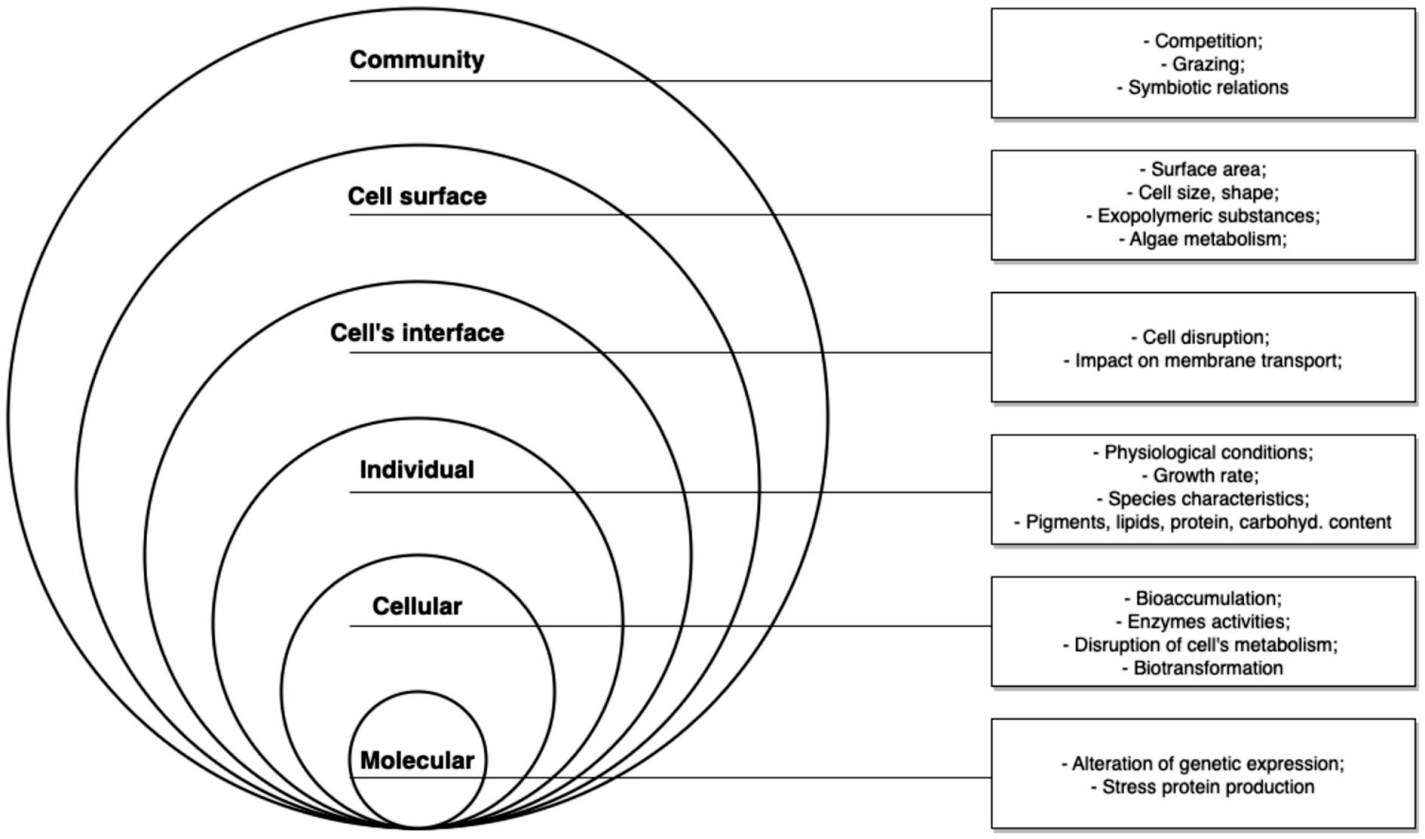
Outcomes of the main question categorized by ecological and biogeochemical parameters

The results concerning algae as biomonitors of pollution in estuaries will be examined, including but not limited to those illustrated in Fig. 1.

## Methods

The systematic map will follow the Collaboration for Environmental Evidence Guidelines and Standards for Evidence Synthesis in Environmental Management (CEE, version 5.1 [6]) and it conforms to ROSES reporting standards [10] (see Additional file 1).

### Search for articles

#### Search terms and languages

Searches will be performed using search terms exclusively in English or Portuguese. This search however retrieved articles written in languages other than English or Portuguese and will not be included (see section “Eligibility criteria”). The list of search terms is presented in the next section (see section “Search string”).

#### Search string

A combination of search terms was obtained based on [18] that performed the same approach for coral reefs after a scoping exercise using ‘litsearchR’ package as Web Of Science format:

TS = ((alga* OR seaweed OR macroalga* OR microalga* OR phytoplankton) AND (estuar* OR coastal OR bay) AND ( contamin* OR toxicant$ OR pollut* OR "industrial discharge$" OR effluent$ OR runoff$ OR sewage OR eutrophication OR wastewater OR nutrient$ OR pesticide$ OR "antifouling agent$" OR biocide$ OR metal$ OR organochlorine$ OR "petroleum product$" OR solvent$ OR PCB$ OR PAH$ OR pharmaceutical$ OR "personal care product$" OR drug$ OR "UV filter$" OR microplastic$ OR nanoparticle$ OR "endocrine disrupting compound$" OR "perfluorinated compound$" OR hydrocarbon$ OR "oil spill$" OR phthalate$ OR "polyfluoroalkyl substances" OR PFAS) AND (biomass OR community OR chemistry OR uptake OR productivity OR biomonitor* OR monitor* OR bioindicator* OR detection OR growth)).

#### Estimating the comprehensiveness of the search

To evaluate the comprehensiveness of the search string, we utilized a test list of 15 papers considered relevant by the review team to address our question, encompassing a variety of pollutants (Additional file 2). These articles cover a diverse range of chemicals and are considered highly relevant to answering our research question. The adjusted search strategy identified all articles from the test list. This finding suggests that our search strategy is highly comprehensive in capturing the relevant literature on the impact of pollution on algae in estuaries.

#### Bibliographic databases

The Federal Fluminense University institutional subscription will be used to search two bibliographic databases: Web of Science Core Collection and Scopus. The searches will include TS (i.e., title, abstract, author keywords) for WoSCC and TITLE-ABS-KEY ('Title, Abstract, Keyword') for Scopus.

### Internet searches

Additional searches of literature will be performed using Google Scholar.

The search string developed during the scoping exercise on WoSCC database will be adapted to fit the search facilities of these search engines (Additional file 3). Searches will be performed on titles, then the results will be sorted by relevance, and the first 400 hits will be extracted. Results from Google Scholar will be extracted using the software Publish or Perish version 8.12.4612 (12 March 2024) (https://harzing.com/resources/publish-or-perish, accessed 30 March 2024). Additionally, we will also adapt the search to look for theses and dissertations in the ProQuest Dissertations and Theses (https://search.proquest.com/, Publicly Available Content Database), and the Open Access Theses and Dissertations (https://oatd.org/). Searches will be performed on titles, sorted by relevance and extract the first 100 hits. Grey literature on two Brazilian online repository of thesis (in Portuguese) will also be done (https://catalogodeteses.capes.gov.br/catalogo-teses/#!/ and http://bdtd.ibict.br/). An adapted search string will also be applied in the Ecotox Knowledgebase (https://cfpub.epa.gov/ecotox/).

### Assembling and managing search results

The results of all searches will be merged and duplicates will be treated using the CADIMA online tool for systematic reviews [12]. The map will be managed with Microsoft Excel software.

### Article screening and study eligibility criteria

#### Screening process

Articles will be screened for eligibility in two successive stages: first on titles and abstracts, and second on full-texts. Titles and abstracts will be imported to CADIMA (https://www.cadima.info/index.php), which is an online evidence synthesis tool. Duplicates are removed and publications evaluated. Articles with unclear eligibility status during title/abstract screening will be included for full text screening. The list of articles with unclear eligibility status after completion of full-text screening will be provided with an explanation of why they could not be classified. Articles without an abstract and retained based on title screening will directly be screened on their full-text. Screening will independently be performed by all reviewers. All reviewers will evaluate articles based on their titles and abstracts. The statistical coefficient Cohen's kappa is calculated to evaluate the level of agreement between reviewers. If the kappa index suggests inconsistency (k < 0.5), the discrepancies will be discussed and resolved through changing inclusion/exclusion criteria and the process repeated. The publications will then be evaluated based on their full text by one reviewer. A subset of 10% of the publications will be assessed by a second reviewer. Again, after kappa index evaluation, any discrepancies will be discussed and resolved. Articles excluded at the second stage will be provided in an appendix at full text, with the reasons for exclusion.

### Eligibility criteria

At each stage, the eligibility of articles will be assessed using the criteria displayed in Table 1.

**Table 1:** Eligibility criteria

Include	Exclude
Population	
All estuarine algal species	Fishes, invertebrates Studies not conducted in estuaries (e.g. Freshwater, marine environments, laboratory)
Exposure	
All natural, geogenic and synthetic chemicals coming from human activities	Studies assessing the impact of chemicals coming from natural sources (e.g. coastal upwelling) Studies assessing the impact of sedimentation per se or of physical disturbances on algae Marine debris, macro-plastics Studies assessing the impact of human activities (e.g. river discharge, distance to a dump or to an industrial effluent source, tourism) on algae without reference to a chemical
Comparator	
Populations unexposed to chemicals Populations prior to exposure to chemicals Populations exposed to a range of concentrations/levels of chemicals Populations exposed for different periods of time to chemicals	
Outcome	
All outcomes related to algae, from molecular level (e.g. gene expression, enzyme activities) to community level (e.g. symbiotic relations, species richness) (Fig. 1) Evidence of concentration or accumulation/uptake of chemicals in the population studied. Bioaccumulation potential with concentration or uptake rate impacts on algal community, algal growth rate, or abundance	
Language	
All articles written in English or Portuguese	
Type of document	
Journal articles, conference or meeting abstract, poster, book chapter, report, conference proceeding, Ph.D. or M.Sc. thesis	Presentation, editorial material, letter or news items
Type of content	
In-situ studies	Reviews and meta-analyses, modeling studies without experimental data, laboratory exposure experiments, ex situ studies

The publications must fulfill the following criteria to be included: Relevant subjects: brackish or estuarine coastal regions. Marine, or freshwater lakes or rivers will not be considered in this review. Relevant types of exposure: algal presence and characteristics. This includes any estuarine algal species (e.g., red, green or brown genera) exposed to all natural and synthetic chemicals coming from human activities. Relevant types of comparator: comparing community through a period of exposure to contaminants, comparing algal tissue exposed to chemicals and population prior to exposure, comparing algae exposed to a range of concentrations/levels of chemicals. Relevant types of outcome: All outcomes related to algae, from molecular level (e.g. gene expression, enzyme activities) to community level (e.g. symbiotic relations, species richness). Also, any measures of concentration, accumulation/uptake of chemicals in the studied population. Different impacts on algal community, algal growth rate, or abundance will also be considered. Relevant types of study: All primary field (in-situ) studies—not reviews, modeling, laboratory-based studies since these do not answer the question (primary studies reported within reviews and meta-analyses will be considered for eligibility). The studies may be from any year (no cut-off point) until the present (2025). Language: full-texts written in English or Portuguese, only. The list of articles rejected at full-text screening will be provided with their reasons for exclusion. Reviews and meta-analyses will be excluded but those eligible according to the Population-Exposure-Comparator-Outcome criteria will be listed in a separate file to make them easily accessible for possible further use.

A flow diagram following the ROSES framework will be created to document the screening results. Articles that were excluded based on title and abstract will be listed in one file, while a separate file will document articles excluded at full text along with explanations for their exclusion. Any departures from this procedure will be disclosed and described.

### Study validity assessment

No critical appraisal of study will be performed for the systematic map. However, data coding will include aspects of study design.

### Data coding strategy

For all studies included in the map, a detailed CodeBook of variables will be available using a Microsoft Excel sheet, with exhaustive information made available (Additional file 4). This documentation will encompass bibliographic details such as unique identifier, source, title, authors, journal, year, DOI, language, and publication type. It will also include a general description of the study detailing publication content, the country of origin, and location by latitude and longitude. The dataset will describe the study population in terms of taxon and taxon level, the nature of exposure as mentioned by the original authors and as categorized by the review team, and the types of outcomes.

The data coding process will be informed by an a priori specified CodeBook (refer to Additional file 4) and will be conducted by at least two independent reviewers. The codebook used for assessing evidence of chemical impacts on coral reefs by [18] will be a reference for this process. The coding system streamlines critical appraisal of algal biomonitoring studies by categorizing them across key dimensions. It classifies research based on publication type (e.g., journal article, thesis), geographic scope, pollutant focus (e.g., hydrocarbons, pesticides, heavy metals), experimental setting (in situ or ex situ), and the level of biological organization studied (from molecular to community).

### Study mapping and presentation

We will produce a database (Microsoft Excel sheet) of all included studies and their coded data and will be cross validated between reviewers. This database will be open access and available as an appendix to the systematic map publication. In the map report, a narrative synthesis approach with descriptive statistics, tables and figures will be used to represent the geographical distribution of the included studies as well as their frequencies in the categories specified in the CodeBook. A matrix showing the distribution and frequency of included study into types of exposure and types of outcomes will be computed. The types of exposure and outcomes a priori defined in the CodeBook will be used, but new types may emerge during the meta-coding process. The matrix will be plotted as a heat map to visually identify potential knowledge gaps and knowledge clusters. We will thus identify the clusters(s) for which a full synthesis of evidence (systematic review) should be possible.

## Supplementary Information

Additional file 1.

Additional file 2.

Additional file 3.

Additional file 4.

## Data Availability

All data generated or analyzed during this study are included in this published article and its supplementary information files.
