# Supplementary material for: What evidence exists on the effectiveness of algae as biomonitors of pollution in estuaries? A systematic map protocol
Source: Environ Evid. 2025 Nov 13;14:23. doi: 10.1186/s13750-025-00378-1 (PMC12616895; doi:10.1186/s13750-025-00378-1)
Supplement: Supplementary file 3 — Additional file 3. [file 13750_2025_378_MOESM3_ESM.docx]

**Additional file 3: Search strings.** Search strings that will be used for searching in publication databases and search engines

- **Web Of Science Core Collection**

TS=((alga* OR seaweed OR macroalga* OR microalga* OR phytoplankton) AND (estuar* OR coastal OR bay) AND ( contamin* OR toxicant$ OR pollut* OR "industrial discharge$" OR effluent$ OR runoff$ OR sewage OR eutrophication OR wastewater OR nutrient$ OR pesticide$ OR "antifouling agent$" OR biocide$ OR metal$ OR organochlorine$ OR "petroleum product$" OR solvent$ OR PCB$ OR PAH$ OR pharmaceutical$ OR "personal care product$" OR drug$ OR "UV filter$" OR microplastic$ OR nanoparticle$ OR "endocrine disrupting compound$" OR "perfluorinated compound$" OR hydrocarbon$ OR "oil spill$" OR phthalate$ OR "polyfluoroalkyl substances" OR PFAS) AND (biomass OR community OR chemistry OR uptake OR productivity OR biomonitor* OR monitor* OR bioindicator* OR detection OR growth))

- **SCOPUS**

TITLE-ABS-KEY=((alga* OR seaweed OR macroalga* OR microalga* OR phytoplankton) AND ( estuar* OR coastal OR bay ) AND ( contamin* OR toxicant$ OR pollut* OR "industrial discharge$" OR effluent$ OR runoff$ OR sewage OR eutrophication OR wastewater OR nutrient$ OR pesticide$ OR "antifouling agent$" OR biocide$ OR metal$ OR organochlorine$ OR "petroleum product$" OR solvent$ OR pcb$ OR pah$ OR pharmaceutical$ OR "personal care product$" OR drug$ OR "UV filter$" OR microplastic$ OR nanoparticle$ OR "endocrine disrupting compound$" OR "perfluorinated compound$" OR hydrocarbon$ OR "oil spill$" OR phthalate$ OR "polyfluoroalkyl substances" OR pfas ) AND ( biomass OR community OR chemistry OR uptake OR productivity OR biomonitor* OR monitor* OR bioindicator* OR detection OR growth))

- **Google Scholar (six search strings of less than 256 characters were used)**

S1: (alga* OR seaweed OR macroalga* OR microalga* OR phytoplankton) AND (estuar* OR coastal OR bay) AND (contamin* OR pollut* OR metal* OR pesticide* OR toxicant$ OR effluent$ OR runoff$) AND (biomonitor* OR monitor* OR growth OR biomass OR community OR detection)

S2: (macroalga* OR microalga* OR phytoplankton OR alga*) AND (estuar* OR bay OR coastal) AND (eutrophication OR nutrient* OR wastewater OR sewage OR runoff$) AND (biomass OR productivity OR uptake OR chemistry OR detection)

S3: (alga* OR seaweed OR phytoplankton OR macroalga*) AND (coastal OR bay OR estuar*) AND ("industrial discharge$" OR effluent$ OR runoff$ OR sewage OR wastewater) AND (community OR chemistry OR detection OR biomonitor* OR monitor*)

S4: (alga* OR seaweed OR phytoplankton) AND (estuar* OR coastal OR bay) AND (pharmaceutical$ OR "personal care product$" OR drug$ OR “UV filter$” OR metal$) AND (biomonitor* OR monitor* OR bioindicator* OR detection OR chemistry)

S5: (seaweed OR macroalga* OR microalga* OR alga*) AND (coastal OR estuar* OR bay) AND (microplastic$ OR nanoparticle$ OR PFAS OR "perfluorinated compound$" OR metal$) AND (uptake OR growth OR biomass OR productivity)

S6: (phytoplankton OR alga* OR seaweed OR macroalga*) AND (bay OR estuar* OR coastal) AND ("endocrine disrupting compound$" OR "oil spill$" OR hydrocarbon$ OR phthalate$) AND (biomass OR productivity OR community OR chemistry)

Some search items could not be included in the Google Scholar search: "industrial discharge$", run-off, waste-water, "industrial product$", "consumer product$", "household product$", "biocidal product$", anti-foul*, "flame retardant$", "perfluorinated compound$", "personal care product$", nano-particle$, "endocrine disrupt*", "organic compound$", "transformation product$", "degradation product$", "UV filter$", "ultraviolet filter$".
