# Supplementary material for: What evidence exists on the effectiveness of algae as biomonitors of pollution in estuaries? A systematic map protocol
Source: Environ Evid. 2025 Nov 13;14:23. doi: 10.1186/s13750-025-00378-1 (PMC12616895; doi:10.1186/s13750-025-00378-1)
Supplement: Supplementary file 1 — Additional file 1. [file 13750_2025_378_MOESM1_ESM.pdf]

Item	Section / sub-	Topic	Description	Further explanation	Checklist/Meta-dat	Author response	Comments
1	Title	Title	The title must indicate that it is a systematic map protocol, and must indicate if it is an update/amendment: e.g. "A systematic map update protocol…".	The title should normally be the same or very similar to the review question.	Meta-data	What evidence exists on the effectiveness of algae as biomonitors of pollution on estuaries? A systematic map protocol	
2	Type of review	Type of review	Select one of the following types of review: systematic map, systematic map update, systematic map amendment	See CEE Guidance on systematic mapping [1], and on amendments and updates [2]	Meta-data	systematic map	
3	Authors contacts	Authors contacts	The full names, institutional addresses, and email addresses for all authors must be provided.		Checklist	Yes	
4	Abstract	Structured summary	Abstract must not exceed 350 words and must include two sections 1) Background, the context and purpose of the review, including the review question; 2) Methods, how the review will be conducted and the outputs that are expected (specifically mention search strategy, inclusion criteria, critical appraisal, data extraction and synthesis).		Checklist	Yes	
5	Background	Background	Describe the rationale for the review in the context of what is already known. Protocol must indicate why this study was necessary and what it aims to contribute to the field.	A theory of change and/or conceptual model can be presented that links the intervention or exposure to the outcome.	Checklist	Yes	
6	Stakeholder engagement	Stakeholder engagement	The planned/actual role of stakeholders throughout the review process (e.g. in the formulation of the question) must be described and explained (using a broad definition of 'stakeholder', including e.g. researchers, funders and other decision-makers; see [3])		Checklist	Yes	
7	Objective of the review	Objective	Describe the primary question and secondary questions (when applicable).	The primary question is the main question of the review. Secondary questions are usually linked to sources of heterogeneity (effect modifiers).	Checklist	Yes	
8		Definitions of the question components	Break down and summarise question key elements e.g. population, intervention(s)/exposure(s), comparator(s), and outcome(s).	For other question types see [4,5]	Meta-data	Components of the primary question<br>Population: algal species in estuaries<br>Exposure: types and concentration of natural and human-produced chemical substances<br>Comparator: population not exposed to pollution; population prior to pollution exposure/ population exposed to different concentrations of pollution<br>Outcome: All outcomes related to estuarine algae being used as biomonitors of pollution, from molecular to community levels. The outcomes related to algae as biomonitors of pollutants in estuaries will be considered including but not restricted to those presented in Figure 1.	
	Methods						
9	Searches	Search strategy		Details regarding search strategy testing should be provided.	Checklist	Yes	
10		Search string	Provide Boolean-style full search string and state the platform for which the string is formatted (e.g. Web of Science format)		Meta-data	(Web Of Science format)<br>TS = ((alga* OR seaweed OR macroalga* OR microalga* OR phytoplankton) AND (estuary OR estuaries OR estuarine) AND (contamin* OR toxicant$ OR pollut* OR "industrial discharge$" OR "sewage effluent$" OR "terrestrial runoff$" OR "agricultural runoff$" OR sewage OR eutrophication OR wastewater OR sedimentation OR nutrient$ OR pesticide$ OR herbicide$ OR insecticide$ OR fungicide$ OR "antifouling agent$" OR "non-agricultural biocide$" OR metal$ OR heavy metal OR organochlorine$ OR petrochemical$ OR solvent$ OR hormone$ OR "transformation product$" OR "degradation product$" OR byproduct$ OR "flame retardant$" OR detergent$ OR antibiotic$ OR "oil product$" OR dispersant$ OR "organic compound$" OR PCB$ OR PAH$ OR pharmaceutical$ OR "personal care product$" OR "UV filter$" OR microplastic$ OR nanoparticle$ OR "endocrine disrupting compound$" OR "perfluorinated compound$" OR hydrocarbon$ OR "oil spill$" OR phthalate$ OR biocide$) AND (biomonitor* OR detection OR monitor*)).	
11		Languages – bibliographic databases	List languages to be used in bibliographic database searches.		Meta-data	English	
12		Languages – grey literature	List languages to be used in organizational websites searches and web-based search engines.		Meta-data	English	
13		Bibliographic databases	Provide the number of bibliographic databases to be searched.		Meta-data		2
14		Web – based search engines	Provide the number of web – based search engines to be searched.		Meta-data		1
15		Organisational websites	Provide the number of organisational websites to be searched.		Meta-data		0
16		Estimating the comprehensiveness of the search	Describe the process by which the comprehensiveness of the search strategy was assessed (i.e. list of benchmark articles).		Checklist	Yes	Benchmark of 15 articles
17		Search update	Describe any plans to update the searches during the conduct of the review.	Optional. A search update is good practice if original searches were performed more than two years prior to review completion.	Checklist	n/a	
18	Article screening and study inclusion criteria	Screening strategy	Describe the methodology for screening articles/studies for relevance/eligibility.		Checklist	Yes	Screening in two stages: first on title/abstract and then on full text.
19		Consistency checking	Describe clearly the process for checking consistency of decisions including the levels at which consistency checking will be undertaken and estimated proportion of articles/studies that will be screened and checked for consistency by two or more reviewers (e.g. Titles (10%), abstracts (10%), full text (10%)).		Checklist	Yes	10% of articles checked for consistency of decisions for title/abstract screening, 5% for full text screening
20		Inclusion criteria	Describe the inclusion criteria used to assess relevance of identified articles/studies. These must be broken down into the question key elements (e.g. relevant subject(s), intervention(s)/exposure(s), comparator(s), outcomes, study design(s)) and any other restrictions (e.g. date ranges or languages).		Checklist	Yes	
21		Reasons for exclusion	State that you will provide a list of articles excluded at full text with reasons for exclusion.		Checklist	Yes	Stated at the end of the "Article screening and study eligibility criteria" section
22	Critical appraisal	Critical appraisal strategy	Describe here the method you propose for critical appraisal of study validity (including assessment of individual studies and the evidence base as a whole).	Optional	Checklist	n/a	No critical appraisal of study will be performed for the systematic map
23		Critical appraisal used in synthesis	Describe how the information from critical appraisal will be used in synthesis.	Optional	Checklist	n/a	
24		Consistency checking	Describe how repeatability of critical appraisal of study validity will be tested.	Optional	Checklist	n/a	
25	Data extraction	Meta-data extraction and coding strategy	Describe the method for meta-data extraction and coding for studies (potentially providing forms/data sheets (ideally piloted), list if variables to be extracted as meta-data and those that will be coded).		Checklist	Yes	Code Book provided in Additional file 4
26	Data synthesis and presentation	Narrative synthesis strategy	Describe methods to be used for narratively synthesising the evidence base in the form of descriptive statistics, tables (including SM database) and figures.	Vote-counting (tallying of studies based on the direction or significance of their findings) must be avoided. May include a summary of the outputs of critical appraisal of the evidence base as a whole (if planned to be performed in SM).	Checklist	Yes	
27		Knowledge gap and cluster identification strategy	Describe the methods to be used to identify and/or prioritise key knowledge gaps (unrepresented or underrepresented subtopics that warrant further primary research) and knowledge clusters (well-represented subtopics that are amenable to full synthesis via systematic review).		Checklist	Yes	Specifically, a matrix showing the distribution and frequency of included study into types of exposure and types of outcomes will be computed
28		Demonstrating procedural independence	Describe the role of systematic reviewers (who have also authored articles to be considered within the review) in decisions regarding inclusion or critical appraisal of their own work.	Reviewers who have authored articles to be considered within the review should be prevented from unduly influencing inclusion decisions, for example by delegating tasks appropriately.	Checklist	Yes	Described at the end of the "Screening process" section
29	Declarations	Competing inter	Describe of any financial or non-financial competing interests that the review authors may have.		Checklist	Yes	Two authors declared potential competing interests.

Reference

[1] James, K.L., Randall, N.P. and Haddaway, N.R., 2016. A methodology for systematic mapping in environmental sciences. Environmental Evidence, 5(1), p.7.

[2] Bayliss, H.R., Haddaway, N.R., Eales, J., Frampton, G.K. and James, K.L., 2016. Updating and amending systematic reviews and systematic maps in environmental management. Environmental Evidence, 5(1), p.20.

[3] Haddaway, N.R., Kohl, C., da Silva, N.R., Schiemann, J., Spök, A., Stewart, R., Sweet, J.B. and Wilhelm, R., 2017. A framework for stakeholder engagement during systematic reviews and maps in environmental management. *Environmental Evidence* , *6* (1), p.11.

[4] Collaboration for Environmental Evidence. 2018. Guidelines and Standards for Evidence synthesis in Environmental Management. Version 5.0. www.environmentalevidence.org/information-for-authors.

[5] Leeds Institute of Health Sciences. https://medhealth.leeds.ac.uk/info/639/information_specialists/1500/search_concept_tools. Accessed 12/11/2017.
